# Exploring whether teaching activity is a way to improve GPs' satisfaction and wellbeing: a cross-sectional study

**DOI:** 10.3399/bjgpopen20X101038

**Published:** 2020-05-27

**Authors:** Blandine Mooser, Nicolas Senn, François Heritier, Christine Cohidon

**Affiliations:** 1 University of Lausanne, 27213, Lausanne, Switzerland; 2 Center of General Medicine and Public Health, Department of Family Medicine, Medecin Associé, Lausanne, Switzerland

**Keywords:** general practitioners, primary health care, teaching activity, loss of meaning in work, job satisfaction

## Abstract

**Background:**

GP dissatisfaction and stress at work have been a growing domain of interest for several decades. However, few studies have focused on positive predictors of GPs’ satisfaction and wellbeing. The diversity of activities could be one area that could be explored to aid job satisfaction.

**Aim:**

The aim of this study was to investigate the association between the meaning in GPs' work and medical teaching activity.

**Design & setting:**

This is a secondary analysis of the Swiss data of the QUALICOPC study, a multicentric European-wide study, investigating primary care quality, costs, and equity.

**Method:**

A total of 199 GPs in Switzerland answered a 60-question postal questionnaire. Descriptive and multivariate logistic regression analyses were performed using Stata (version 15). A focus group with six GPs gave qualitative data to help interpret the results.

**Results:**

Thirty-one per cent of GPs reported a loss of meaning in their work. In multivariate analyses, loss of meaning was lower in GPs with teaching activity (odds ratio [OR] = 0.50; 95% confidence interval [CI] = 0.27 to 0.90). In addition, loss of meaning was associated with late hospital discharge letter reception time (OR = 2.28; 95% CI = 1.20 to 4.35 if ≥15 days) and an administrative overload (OR = 4.18; 95% CI = 2.04 to 5.58). For GPs in the focus group, medical teaching occurred mostly because of intrinsic motivations.

**Conclusion:**

Loss of meaning in GPs' work was lessened with teaching activity. Therefore, encouraging a GP practice that is varied in its activities may encourage GP satisfaction. Ultimately, job satisfaction may impact patient quality of care and this study offers some insight on how to improve work satisfaction for the next generation of GPs.

## How this fits in

A variety of activities could be a positive predictor of job satisfaction among GPs. However, among these activities, teaching activity is generally rarely investigated. This study revealed a strong association between teaching activity and a lower loss of meaning in work among GPs. Teaching activity could be an interesting perspective to promote the profession of GPs.

## 

## Introduction

GPs' dissatisfaction and stress at work have been a growing domain of interest for several decades.^1,2^ The consequences of dissatisfaction and stress have been extensively studied in terms of: (1) the harmful effects on individual mental health such as burnout and depressive syndromes; (2) crisis in the recruitment and retention of GPs;^3,4^ and (3) potential repercussions on patients’ quality of care.^5–7^ The origins of dissatisfaction and stress at work in GPs have also been investigated; for example, heavy workload, especially administrative tasks; fragmentation of activities and time pressure; regulation and control of practices; and increases in patient demands have been pointed out.^8–11^ Exposure to all of these occupational pressures, but in particular administrative overload, could lead to a loss of meaning at work (or brownout in some occupational sectors,^12^ which occurs when there is a discrepancy between the expected job and the reality of it). Although there is much research into the causes and consequences of GPs’ dissatisfaction at work, studies considering positive predictors of GPs' satisfaction and wellbeing at work are fewer.^13^ In parallel, research regarding interventions to improve GPs’ wellbeing needs to be developed.^14^ Many authors have reported that a variety of activities could be a positive predictor of satisfaction among GPs.^2,3,13,15,16^ These various activities could include both clinical and non-clinical activities at the practice. Among these, teaching activity is generally rarely mentioned,^17–20^ but being a mentor may be considered by physicians as a valued part of the job.^15^ Even Hippocrates considered the teaching activity of a doctor essential for the profession. In the UK, the General Medical Council has recognised that all doctors have teaching responsibilities.^21^ According to Harden and Laidlaw, everyone is capable of being a good teacher with a range of technical skills, an understanding of basic educational principles, an enthusiasm and passion for teaching, and a commitment to evaluating and improving their own teaching.^22^

The aim of this study was to investigate the predictors of loss of meaning in work among GPs and, in particular, to describe the association with teaching activity.

## Method​

### Study design

This study is a secondary analysis of the Swiss data of the QUALICOPC study. Directed by the Netherlands Institute for Health Services Research (NIVEL), the QUALICOPC study aimed to compare the global performance of healthcare systems according to quality, costs, and equity of primary care in 34 countries, including Switzerland.^23^

The QUALICOPC questionnaires were developed in several steps. A framework of topics of interest was created and a search conducted on existing questionnaires.^24^ Next, the questionnaires were translated into the national languages and distributed. All questionnaires have a uniform design, a closed answering format, and are anonymous.^23^ Ethical approval for the study in Switzerland was acquired.^25^

### ​Selection of study participants

In Switzerland, a random sample of 2027 GPs was drawn from two primary care physicians’ associations. Ten per cent of those GPs agreed to be recruited in the long term to form the Swiss Primary Care Active Monitoring (SPAM) network.^24^ Of those, almost 100% participated in the present QUALICOPC study, for a total of 199 GPs. The representativeness of the sample in terms of sex, rural or urban setting, and age was considered satisfactory when cross-checked against national statistics.^26^ In Switzerland, the research data were collected in 2012.

### ​Questionnaire content

The 60-question postal questionnaire investigated the organisation of the GP practice. Sociodemographic characteristics, namely sex, age, country of birth, and practice location, were also investigated. Other questions in the survey explored organisational practice attributes, and meeting face to face with other healthcare professionals, either professionally or socially. Practice organisation was characterised by the GP working alone, in shared premises with other GPs or with medical specialists, or with other disciplines. Exposure to heavy administrative workload and the feeling of respect related to the occupation of the GP were explored (4-point Likert scale, from ‘strongly disagree’ to ‘strongly agree’). Having other activities besides clinical activity at the practice was assessed with the following question: 'Besides your work as a GP in this practice, do you have any other paid professional activities?' (Possible answers were: no; yes, as a physician for privately paying patients; yes, in a residential setting [for example, nursing home, prison]; yes, as a company doctor; yes, in teaching or medical education; yes, other). Exposure to loss of meaning in the work was explored via the following question: 'To what extent do you agree with the following statements? I feel that some parts of my work do not really make sense.' This had four possible answers, ranging from ‘strongly disagree’ to ‘strongly agree’.

### ​Statistical analyses

First, descriptive analyses determined the frequency of GPs’ exposition to loss of meaning at work and the different potential predictors. Then, multivariate logistic regression analyses explored multiple predictors of loss of meaning simultaneously. The dependent variable was the loss of meaning of the work, while the independent variables were the sociodemographic characteristics (namely sex, age, location of practice, linguistic zone), GP practice organisation and attributes, GP psychosocial exposure, and having other work activities. Regarding this aspect, the global variable was first tested, that is, 'having other activities versus no other activities', and then specific analyses were performed for each kind of activity. Variables that were statistically significant with *P*<0.2 (to allow for a greater number of explorations) in the univariate associations were introduced one at a time in the multivariate model.

### ​Focus group

Six GPs, currently practising and members of the Department of General Medicine, were solicited to allow a better understanding of the term 'loss of meaning in the work' that was used in the QUALICOPC questionnaire, and to give a glimpse into their real-life work perspective. They were asked the following: what was their personal definition of 'loss of meaning of the work'; what was their opinion on the association between feeling satisfied and teaching; and what were their thoughts on in-practice teaching and university teaching. Their answers were summarised and transcribed.

## Results

The sociodemographic and practice characteristics of the GPs are described in Table 1. GPs in this sample were predominantly male and the age ranged from 35 to 74 years, with a median age of 56 years. The distribution between rural and urban practice location and solo and group practice was equally split. About 80% of GPs responded that their work was overloaded with administration and 67% reported that being a GP is a well-respected job. About one GP out of three (31%) complained that some parts of their work do not really make sense (Table 2).

**Table 1.** Characteristics of participating GPs

**Sociodemographic characteristics**	*n* (%)^a^
Sex	
Male	155 (78)
Female	44 (22)
Age, mean (SD)	55.0 (8.0)
Practice location^b^	
Rural	102 (51)
Urban	95 (48)
Type of practice	
Group practice	104 (52)
Solo practice	95 (48)

*N* = 199. ^a^Unless stated otherwise. ^b^Self-declared. Missing data (*n* = 2). SD = standard deviation

**Table 2.** Characteristics of the practices' organisation and GPs’ loss of meaning in work

**Practice organisation**	*n* (%)^a^
Total working hours per week, mean (SD)	46.6 (11.6)
Duration of consultation, minutes, mean (SD)	19.6 (5.8)
Usual time necessary for receiving hospital discharge, days^b^	
1–4	60 (30)
5–14	80 (40)
15–30	38 (19)
>30	14 (7)
Being GP as unique work activity	67 (34)
Teaching activity	61 (31)
Number of face-to-face patient contacts in a day, mean (SD)	24.0 (8:1)
Number of email patient contacts in a day, mean (SD)	1.5 (2.9)
Number of night on-call duties in the past 3 months, mean (SD)	4.4 (10.5)
Number of weekend on-call duties in the past 3 months, mean (SD)	2.1 (5.3)
Meeting face to face with other GPs	
>1/month	120 (60)
Meeting face to face with hospital specialists	
>1/month	42 (21)
Feeling of lack of respect^c^	
Strongly disagree	12 (6)
Disagree	119 (60)
Agree	53 (27)
Strongly agree	12 (6)
Administrative overload	
Strongly disagree	1 (1)
Disagree	39 (20)
Agree	86 (43)
Strongly agree	73 (37)
Loss of meaning in work^d^	
Strongly disagree	62 (31)
Disagree	75 (38)
Agree	48 (24)
Strongly agree	13 (7)

*N* = 199. ^a^Unless otherwise stated. ^b^Missing data (*n* = 7). ^c^Missing data (*n* = 3). ^d^Missing data (*n* = 1). SD = standard deviation

The results of the univariate and multivariate analyses are described in Table 3. Loss of meaning of work was less prevalent among older GPs in univariate analysis (OR = 0.44; 95% CI = 0.21 to 0.91), but the association did not persist significantly in the multivariate model (OR = 0.52; 95% CI = 0.24 to 1.14; *P* = 0.10). In the same way, loss of meaning of work could be reduced with a higher number of face-to-face contacts a day but the *P* value was borderline (OR = 0.87; 95% CI = 0.73 to 1.04; *P* = 0.14).

**Table 3.** Associations (odds ratios) between sociodemographic and work characteristics and GPs’ loss of meaning in work.

**Covariate**	**Univariate model**(***N* = 199**)	**Final multivariate model**(***N* = 190**)
	*n*	OR	95% CI	OR	95% CI
Sex, female	44	1.15	0.63 to 2.12	0.70	0.35 to 1.39
Age group, years, ref <50 years	49	—	—	1	—
50–60	95	0.85	0.45 to 1.60	0.98	0.50 to 1.91
≥61	55	0.44	0.21 to 0.91	0.52	0.24 to 1.14
Practice location, urban	95	1.30	0.78 to 2.17	—	—
Type of practice, group practice	104	1.02	0.61 to 1.69	—	—
Working hours per week	195	1.00	0.98 to 1.02	—	—
Duration of consultation	196	1.02	0.98 to 1.07	—	—
Discharge information, >15 days	52	2.68	1.46 to 4.92	2.28	1.20 to 4.35
Face-to-face contacts per day^a^	198	0.87	0.75 to 1.02	0.87	0.73 to 1.04
Email patient contacts per day	198	0.98	0.90 to 1.07	—	—
Night on-call duties, *n*	183	1.01	0.98 to 1.03	—	—
Weekend on-call duties, *n*	183	0.96	0.90 to 1.03	—	—
Meeting with other GPs, >1/month	120	0.69	0.41 to 1.17	—	—
Meeting with hospital specialists, >1/month	42	0.88	0.48 to 1.64	—	—
Administrative overload, agree and strongly agree	159	4.53	2.28 to 9.01	4.18	2.04 to 8.58
Feeling of lack of respect, agree and strongly agree	65	3.06	1.73 to 5.40	2.00	1.10 to 3.63
Teaching activity, yes	61	0.52	0.29 to 0.91	0.50	0.27 to 0.90

^a^Unit = five consultations. OR = odds ratio.

Regarding the issue of side activities, the study first tested the global variable, that is, 'having other activities versus no other activities' but the association was not significant and the OR close to 1. Then, specific analyses were performed for the following activities: residential setting doctor, company doctor, and teaching. An association was only found for the last activity: GPs who had teaching activities were significantly less susceptible to perceiving a loss of meaning in their work compared with those without this kind of side job (OR = 0.50; 95% CI = 0.27 to 0.90).

In addition, loss of meaning in the work was also associated with feeling disrespected as a GP (OR = 2.00; 95% CI = 1.10 to 3.63), as well as with long delays for GP receipt of patients’ hospital discharge letters (OR = 2.28; 95% CI = 1.20 to 4.35 if ≥15 days). Finally, loss of meaning was also associated with an administrative overload (OR = 4.18; 95% CI = 2.04 to 5.58).

The focus group shed light on several aspects of GP practices. First, a loss of meaning in work tends to be perceived when GPs *’* *feel like they are losing their freedom* *’*, when they are involved in activities that are not part of the practice of medicine in their opinion, and when they *’* *have to justify themselves* *‘*; for example, when they *’* *need to justify every medical act towards insurances* *‘* or when they *‘* *feel* *”* *i* *nstrumentali* *s* *ed* *”* *by specialists* *’*, other healthcare professionals, or by the healthcare system (for instance, through the role of gatekeeper or in case of defensive medicine). Moreover, the feeling of being buried under administrative tasks also leads to a loss of meaning in the work. Finally, the *’* *discontinuity of information between health* *care providers (especially with hospital physicians)* *’* was mentioned as a source of loss of meaning in the work.

The focus group GPs reported that the association between teaching activity and having a sense of meaning in work was bidirectional. Teaching was a source of satisfaction by increasing work variety, allowing them *’* *to transmit* [their] *knowledge and pride* *‘*, and helping them *’* *find an appropriate work* *–life balance* *‘*. It also allowed them to stay up to date with their medical knowledge. On the other hand, feeling good at work generated *’* *the desire to pass on knowledge* *‘* and made it possible to be a good teacher. Lastly, they pointed out that in-practice teaching and university teaching offered different advantages. In-practice teaching made them *‘* *feel* *at home* *'* and allowed them *’* *to be more creative* *‘*. University teaching was seen to be *‘* *more prestigious* *’*, yet more restrictive.

## Discussion

### Summary

The results reported that about one-third of the Swiss GPs declared that some part of their work did not really make sense. As the focus group GPs stated, this feeling could arise when feeling like they’re straying from the profession they were meant to do, by justifying expenses with insurance companies, filling out paperwork, or being a puppet in specialists’ hands. In addition, this cross-sectional study revealed a strong association between teaching activity and a lower loss of meaning in work among GPs.

### Strengths and limitations

This study has a few limitations. First, its cross-sectional nature prevents evaluation of causality. Its relatively small sample size may also cause a selection bias. Its reliance on self-declared factors may introduce a bias. Moreover, the data is now 7 years old and there is not a more recent study in Switzerland to observe the potential evolution. However, the qualitative studies conducted throughout Europe highlights the importance of teaching for work satisfaction.^15^ Regarding strengths, the sample was nationally representative, the database contained only a few missing values, and the study remains an asset to a country that does not have systematic GP data collection.^25^

### Comparison with existing literature

The potential favourable impact of teaching activity for GPs is an interesting development. Furthermore, this result may not be specific to Swiss physicians. Several studies indicate that GPs appreciate the diversity in their work.^9,16,27–29^ However, a recent study focusing on Swiss GPs showed that their work variety had decreased between 1993 and 2012 since they now do less paediatrics, gynaecology, and so on.^25^ Having side activities, such as teaching*,* could be an answer to this issue. The daily clinical activities are sometimes perceived as too monotonous, especially with patients’ increasing chronic conditions. The presence of a student or a resident is motivating and, through their new point of view and their questioning, may be intellectually challenging. It can also encourage GPs to stay up to date on their medical knowledge. It is also an opportunity for sympathetic and profitable encounters with the new generations of future doctors who are supposed to succeed.^30,31^ GPs often feel a sense of responsibility towards the younger generation. In addition to the medical office teaching activity, there is also a possibility for the motivated GP to teach at a medical school, increasing the variety of activities and so the enjoyment. In this perspective, teaching is a way to reduce the feeling of loneliness sometimes reported by GPs. Indeed, being officially involved in a team of teachers is another way to value the competencies of clinicians. Indeed, these competencies are not only useful for patients, but also for students as they serve as 'knowledge transmitters' officially recognised by their peers. Based on this, faculties and academic family medicine institutions should probably place more value on the identity and activities of family medicine teaching faculty, informal in-practice teaching being equally as important as 'prestigious' ex cathedra lectures.

Regarding the other predictors of loss of meaning in work, the harmful impact of administrative overload on GPs has been pointed out several times in terms of dissatisfaction and burnout.^2,3,10,11,14^ To the authors' knowledge, this is the first time that administrative overload is demonstrated to be associated with a loss of meaning for GPs in their work. In a UK study conducted at the national level, 80% of the 3000 GPs surveyed felt that they were required to do unimportant administrative tasks, preventing their completion of more important ones.^32^ Administrative work may increase over the years, owing to insurance restrictions, hyper-specialisation of medicine, and greater patient list size; therefore, this issue needs to be tackled rapidly.

It is also worth noting that receiving hospital discharges after 4 days (that is, not immediately) was associated with GPs reporting a loss of meaning in their work. Possibly, GPs feel excluded from the medical network when obligated to wait for updates regarding their own patients, making them feel disrespected or underappreciated by specialists. Moreover, having to update their patient’s file when they are removed from the situation may be excessively time-consuming and overwhelming. Therefore, the hospital discharge question at first sight might seem trivial, but in reality, it may be indicative of a larger problem.

### Implications for practice

GPs are facing succession issues throughout western Europe, the UK, and the US, raising questions as to how to take care of the ageing populations and challenging the efficiency of healthcare systems. To make the situation worse, too few medical students wish to become GPs. This is in part because of a poor image of GP work and awareness of stress factors, such as high workload. It may also be because the income of GPs is lower than specialists’ income. Therefore, primary care is quickly becoming a prime target of health research, and GP wellbeing at work is a key issue to retain the workforce. The fact that the loss of meaning in work is reduced among GPs who have a teaching activity offers an interesting perspective to promote the profession.

## Permission to use the QUALICOPC data

In accordance with the contract (INT040-NC24) between the Netherlands Institute for Health Services Research (NIVEL) and the national coordinators of the study, each national coordinator was entitled to use his own national data. Dr Nicolas Senn was the national coordinator for Switzerland. The dataset generated and analysed during the current study is not publicly available, as part of an international study under agreement. However, data could be available from the corresponding author on reasonable request.
